# International Comparisons of Clinical Demographics and Outcomes in the International Society of Pediatric Oncology Wilms Tumor 2001 Trial and Study

**DOI:** 10.1200/GO.21.00425

**Published:** 2022-05-10

**Authors:** Joaquim Caetano de Aguirre-Neto, Beatriz de Camargo, Harm van Tinteren, Christophe Bergeron, Jesper Brok, Gema Ramírez-Villar, Arnauld Verschuur, Rhoikos Furtwängler, Lisa Howell, Daniel Saunders, Oystein Olsen, Aurore Coulomb, Christian Vokuhl, Jan Godzinski, Anne M. Smets, Gordan M. Vujanic, Marry M. van den Heuvel-Eibrink, Norbert Graf, Kathy Pritchard-Jones

**Affiliations:** ^1^ Paediatric Haemato-oncology, Hospital Santa Casa de Belo Horizonte, Belo Horizonte, Brazil; ^2^ Instituto Nacional do Cancer, Research Center, Rio de Janeiro, Brazil; ^3^ Princess Maxima Center for Pediatric Oncology, Utrecht, the Netherlands; ^4^ Centre Léon Bérard, Institut d'Hématologie et d'Oncologie Pédiatrique, Lyon, France; ^5^ Department of Paediatric Oncology and Haematology, Rigshospitalet, Copenhagen, Denmark; ^6^ Pediatric Oncology, Hospital Virgen del Rocío, Sevilla, Spain; ^7^ Service d'hématologie-oncologie Pédiatrique, Hôpital de la Timone, Marseille, France; ^8^ Department of Pediatric Hematology and Oncology, Saarland University Hospital, Homburg, Germany; ^9^ Paediatric Oncology, Alder Hey Children's Hospital NHS Foundation Trust, Liverpool, United Kingdom; ^10^ Paediatric Radiotherapy, The Christie NHS Foundation Trust, Manchester, United Kingdom; ^11^ Radiology, Great Ormond Street Hospital for Children NHS Foundation Trust, London, United Kingdom; ^12^ Service d'Anatomie et Cytologie Pathologiques, Hôpital Armand Trousseau, Paris, France; ^13^ Department of Pathology, University of Bonn, Bonn, Germany; ^14^ Department of Paed. Surgery, Marciniak Hospital, Wroclaw, Poland; ^15^ Radiology, Amsterdam University Medical Centre—AMC, Amsterdam, the Netherlands; ^16^ Department of Pathology, Sidra Medicine, Doha, Qatar; ^17^ UCL Great Ormond Street Institute of Child Health, Developmental Biology and Cancer Research and Teaching Department, University College London, London, United Kingdom

## Abstract

International comparisons of patient demographics, tumor characteristics, and survival can shed light on areas for health care system improvement. The International Society of Pediatric Oncology Wilms Tumor 2001 trial/study registered patients through national clinical study groups in Western Europe and Brazil. This retrospective post hoc analysis of the International Society of Pediatric Oncology Wilms Tumor 2001 database aims to make visible and suggest reasons for any variations in outcomes.

**METHODS:**

All patients with unilateral Wilms tumor (WT), age > 6 months, treated with preoperative chemotherapy as per protocol, and registered between 2001 and 2011 were eligible. Countries were grouped to give comparable case numbers and geographical representation. Cox univariable and multivariable (MVA) statistics were applied, with the German collaborative group (Gesellschaft für Pädiatrische Onkologie und Hämatologie—Austria, Germany, and Switzerland) as reference for hazard ratios for event-free survival (EFS) and overall survival (OS).

**RESULTS:**

A total of 3,176 eligible patients were registered from 24 countries assigned into six groups. Age and histologic risk group distribution were similar across all groupings. The distribution of WT stage varied by country grouping, with 14.9% (range, 11.1%-18.2%) metastatic at diagnosis. Median follow-up was 78.9 months. For localized WT, 5-year EFS varied from 80% (Brazilian group) to 91% (French group; *P* < .0001), retaining significance only for Brazil in MVA (*P* = .001). Five-year OS varied from 89% (Brazilian group) to 98% (French group; *P* < .0001). In MVA, only superior OS in France was significant (*P* = .001). Five-year EFS/OS for stage IV did not vary significantly. High-risk histology and tumor volume at surgery were significantly associated with increased risk of death in MVA for metastatic disease.

**CONCLUSION:**

International benchmarking of survival rates from WT within a large trial/study database has demonstrated statistically significant differences. Clinical interpretation should take account of variation in tumor stage but also treatment factors.

## INTRODUCTION

International comparisons of cancer survival rates can highlight areas for health care improvement.^1^ These include opportunities for earlier diagnosis, reducing variation in how standardized treatments are applied, and revealing differences in tumor biology between populations. Ideally, such comparisons use population-based cancer registry data to avoid any selection bias in the study cohorts.^2^ The disadvantage of using cancer registry data is that they often lack relevant details of patient demographics and tumor characteristics used for clinical risk stratification, treatments given, and if relapse occurred.

**CONTEXT**

**Key Objective**
Is there variation in event-free survival and overall survival for Wilms tumor between countries participating in the same protocol using prenephrectomy chemotherapy?
**Knowledge Generated**
Survival variations between countries and geographic regions exist and may be partially explained by differences in disease burden at diagnosis (tumor stage and volume). Survival variation is most significant in localized Wilms tumor, where therapy is minimized.
**Relevance**
Participation in multinational clinical trials and studies allows benchmarking of prognostic factors between populations with Wilms tumor and emphasizes the importance of central pathology review and quality assurance of risk stratification of postoperative treatment to further improve the success of first-line therapy and avoidance of relapse.

Childhood cancer survival rates vary widely between countries and world regions.^3^ Many factors account for these disparities, including national income status (World Bank country classifications by income level), characteristics of health care systems, accuracy of diagnosis and risk stratification, quality of treatment, supportive care, proportion of patients included in trials, and differences in tumor biology.^4,5^

Clinical trial data sets can be used for such comparisons, particularly in childhood cancers where the clinical community has a history of enrolling a high proportion of all cases into international cooperative group studies. Such within-trial comparative analyses have revealed national differences in tumor volume and stage at diagnosis in Wilms tumor (WT),^6^ neuroblastoma,^7^ and Ewing sarcoma, and in timing and use of radiotherapy and more intensive chemotherapeutic strategies in several childhood cancers (Hodgkin^8^ and Burkitt lymphomas,^9^ Ewing sarcoma,^10^ retinoblastoma,^11^ and germ cell tumors^12^). For a childhood cancer such as WT, where 5-year overall survival (OS) rates approach 90%, understanding such variations may have as great an impact on optimizing treatment and relapse-free survival as introduction of new therapies.

In the International Society of Pediatric Oncology Wilms Tumor 2001 (SIOPWT2001) study, all participating institutions committed to offering registration to all consecutive newly diagnosed children with renal tumors and to apply a standardized diagnostic and risk-stratified treatment protocol. The clinical trial database provides a rich clinical registry for interrogating variation in patient demographics and tumor characteristics, treatments given, and event-free survival (EFS) and OS rates. Hence, we undertook a retrospective post hoc analysis of the SIOPWT2001 database to make visible and suggest reasons for the differences in survival rates observed between some geographical regions and to suggest possible strategies for further improvements in WT management.

## METHODS

### Participants

Patient inclusion criteria for this analysis were all patients with histologically proven stage I-IV unilateral WT, age > 6 months at diagnosis, treated with preoperative chemotherapy before tumor resection, and registered in the SIOPWT2001 study opening in June 2001 until December 31, 2011. This cutoff date was chosen since Grupo Cooperativo Brasileiro para o Tratamento do Tumor de Wilms (GCBTTW) and Children's Cancer and Leukemia Group—United Kingdom, Republic of Ireland, Australia, and New Zealand (CCLG) closed the study on this date to new patient registration, although it continued as a registration study in some other countries.

Twenty-four countries had registered patients meeting these criteria in the SIOPWT2001 study. They were grouped into six cohorts for this analysis, labeled as GPOH (Gesellschaft für Pädiatrische Onkologie und Hämatologie—Austria, Germany, and Switzerland); SFCE (Société Française de lutte contre les Cancers et les leucémies de l'Enfant et de l'adolescent—France); CCLG (Children's Cancer and Leukaemia Group—Australia, Republic of Ireland, New Zealand, and United Kingdom); NOPHO-BSPOH-DCOG (Nordic Society of Paediatric Haematology and Oncology: Denmark, Norway, and Sweden—NOPHO; Belgian Society of Paediatric Haematology Oncology—BSPOH—Belgium; Dutch Children's Oncology Group—DCOG—the Netherlands); GCBTTW (Brazilian Wilms' Tumor Study Group-Brazil); SIOP-OTHER (Argentina, Croatia, Czech Republic, Greece, Italy, Poland, Serbia and Montenegro, Slovak Republic, Slovenia, and the Spanish national group, Sociedad Española de Hematología y Oncología Pediátricas); participating countries are listed in the Data Supplement. Only the SFCE and GCBTTW groups comprised a single country (France and Brazil, respectively).

The cohort analyzed comprised 3,176 patients with localized and metastatic unilateral WT. The median follow-up was 78.9 months.

### Diagnosis, Tumor Staging, and Treatment

The SIOPWT2001 randomized trial and study has been described fully elsewhere.^13^ Nonrandomized patients followed a standardized approach to diagnosis, risk stratification, and preoperative and postoperative treatment according to the protocol recommendations.

In SIOPWT2001, only abdominal ultrasound and chest X-ray (posteroanterior plus lateral view) were mandatory diagnostic investigations, but cross-sectional imaging of the abdomen by computed tomography (CT) or magnetic resonance imaging scan and CT scan for lung or mediastinal nodal metastases were according to national recommendations and practice. Three-dimensional tumor volume was recorded at diagnosis and after completion of preoperative chemotherapy, according to whichever imaging method had been applied.

The preoperative chemotherapy was standardized according to whether metastases were detected at diagnosis: a 4-week regimen of two drugs (actinomycin-D and vincristine [AV]) for cases with localized disease, or a 6-week regimen of three drugs (AV and doxorubicin [AVD]) for cases with metastatic disease, followed by unilateral nephrectomy.

Central review of pathology was highly recommended and was performed in 87.2% of all cases but only in 25% in GCBTTW. The International Society of Pediatric Oncology (SIOP) histologic classification of WT treated with preoperative chemotherapy considers three risk groups, which take account of the relative proportion of viable tumor cells and necrotic or regressive changes: low-risk (100% necrotic), intermediate-risk (epithelial, stromal, mixed, regressive type, and focal anaplasia), and high-risk (diffuse anaplasia and blastemal type).^14^

After nephrectomy, the intensity and duration of postoperative chemotherapy, combined with radiotherapy (if necessary), was determined by tumor histologic risk classification and stage.

The randomized SIOPWT2001 clinical trial compared postoperative chemotherapy with doxorubicin versus no doxorubicin in patients with stage II or III WT with intermediate-risk histology and the reduced therapy experimental group (without doxorubicin) became the new standard of care after March 2011 for this subgroup of patients.

Radiotherapy to the flank/abdomen was recommended for abdominal tumor stage II (only diffuse anaplasia) and III (intermediate-risk and high-risk), and radiotherapy to metastatic sites was recommended for metastases that could not be resected completely, showed incomplete response to chemotherapy, or where the kidney tumor was of high-risk histology, regardless of response.

### Ethical Approval

National and local regulatory and ethical approvals were obtained according to national regulations in all participating countries. Written informed consent for participation was obtained from the parents or legal representatives of the patients and included use of data for secondary analyses. The steering committee of the SIOP Renal Tumor Study Group (SIOP-RTSG) approved the research proposal for the current study, and anonymized data analyses were made available to the researchers through statistical reports generated by data scientists of the SIOP-RTSG office.

### Statistical Analysis

EFS was defined as the time from diagnosis until recurrence or death from any cause, whichever was observed first. OS was the time from diagnosis to last follow-up or until death from any cause. Patients without an event were censored at their time of last follow-up.

Survival distributions were calculated using the Kaplan-Meier technique and compared using the log-rank tests. Hazard ratios and the corresponding 95% CIs were estimated with the use of Cox proportional-hazards models. For the adjusted model (multivariable [MVA] model), all variables from the univariable analysis were included. Global *P* values were derived from the Wald statistic. The proportional hazards assumption was confirmed by visual inspection of the curves.

## RESULTS

### Patient Demographics and Tumor Characteristics

The demographic, tumor characteristics, and diagnosis of 3,176 patients included in this analysis is shown in Table 1. The overall F/M ratio was 1.13 (range, 0.99-1.29). Sex and age distribution was similar across all groups with no statistically significant variation. Stage distribution varied significantly between country groupings, with the highest proportion of stage I disease (53.4%) observed in GPOH and more metastatic disease in CCLG (stage IV: 18.2%). The proportions of tumors classified as low risk, intermediate risk, or high risk showed some variation that did not reach statistical significance, although the SFCE indicated more high-risk tumors (16.7%) than the other groups (11.7%-14.5%). Within the high-risk tumors, diffuse anaplasia was reported in only six of 48 high-risk tumors (12.5%) in the Brazilian group compared with 162 of 436 (38.5%) in all high-risk tumors.

**TABLE 1:**
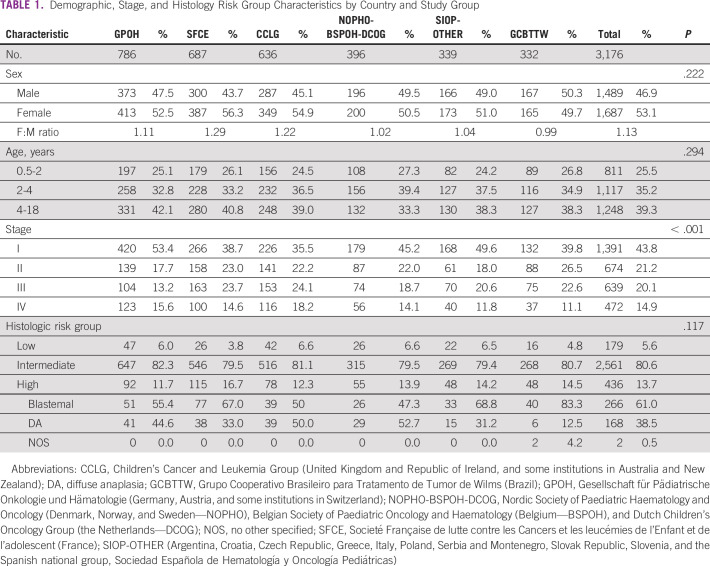
Demographic, Stage, and Histology Risk Group Characteristics by Country and Study Group

Mean tumor volume at diagnosis varied from 464 mL in the GPOH group to 665 mL in the CCLG group (*P* < .001). GCBTTW had the smallest change in tumor volume following preoperative chemotherapy (mean 259 mL), whereas CCLG had the most noticeable shrinkage (mean 356 mL; Data Supplement).

### Treatment

For patients with localized disease, postoperative chemotherapy consisted of only two drugs (AV) or less in 57.7%. Additional doxorubicin, which is included in both the AVD and high-risk regimens used for some risk-stratified postoperative chemotherapy regimens, was used least often in GPOH cases. More than half of the metastatic patients (55.8%) were treated with AVD and 36.5% received the high-risk regimen with four drugs (Table 2). Data on postoperative chemotherapy regimen were missing in 10.4% and 16.5% of all cases with localized tumors and metastatic disease, respectively.

**TABLE 2:**
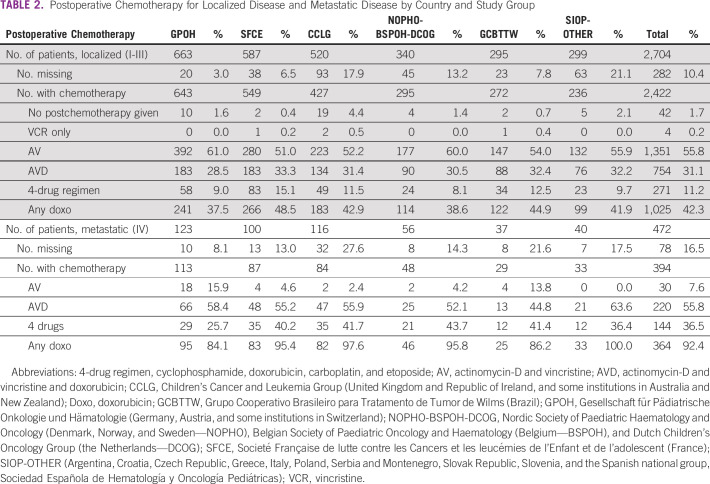
Postoperative Chemotherapy for Localized Disease and Metastatic Disease by Country and Study Group

### Outcomes

**FIG 1:**
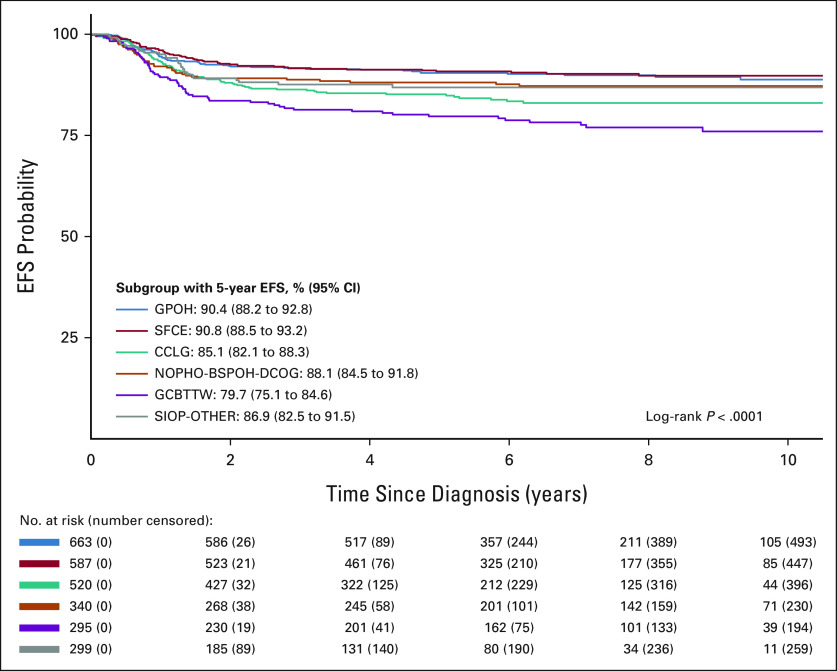
Kaplan-Meier EFS curves for localized disease by country grouping. CCLG, Children's Cancer and Leukemia Group (United Kingdom and Republic of Ireland, and some institutions in Australia and New Zealand); EFS, event-free survival; GCBTTW, Grupo Cooperativo Brasileiro para Tratamento de Tumor de Wilms (Brazil); GPOH, Gesellschaft für Pädiatrische Onkologie und Hämatologie (Germany, Austria, and some institutions in Switzerland); NOPHO-BSPOH-DCOG, Nordic Society of Paediatric Haematology and Oncology (Denmark, Norway, and Sweden—NOPHO), Belgian Society of Paediatric Oncology and Haematology (Belgium—BSPOH), and Dutch Children's Oncology Group (the Netherlands—DCOG); SFCE, Societé Française de lutte contre les Cancers et les leucémies de l'Enfant et de l'adolescent (France); SIOP-OTHER (Argentina, Croatia, Czech Republic, Greece, Italy, Poland, Serbia and Montenegro, Slovak Republic, Slovenia, and the Spanish national group, Sociedad Española de Hematología y Oncología Pediátricas).

The 5-year EFS and OS for the whole cohort were 85.6% (95% CIs, 84.3 to 86.9) and 93.0% (95% CIs, 92.1 to 93.9), respectively. In Kaplan-Meier analyses, EFS for localized disease (stages I-III, all WT subtypes) varied by country grouping (*P* < .0001, Fig 1 and Data Supplement). For metastatic disease, the country group EFS and OS variations did not reach statistical significance (Data Supplement, *P* = .33, and Data Supplement, *P* = .065). Crude and adjusted hazard ratio with 95% CIs for country/group, age, sex, stage, histologic risk, and tumor volume at surgery for localized and metastatic disease are shown in Tables 3 and 4, respectively. For localized disease, CCLG and GCBTTW showed a higher risk of relapse on univariable analysis, but this retained significance only for GCBTTW in MVA (*P* = .001). Other independent prognostic factors for EFS were age (*P* < .0001), stage III (*P* = .001), high-risk histology group (*P* < .0001), and tumor volume at surgery (*P* < .0001). For metastatic disease, only high-risk tumors (*P* < .001) and tumor volume at surgery (*P* = .001) were associated with increased risk for relapse, with no significant associations with country grouping.

**TABLE 3:**
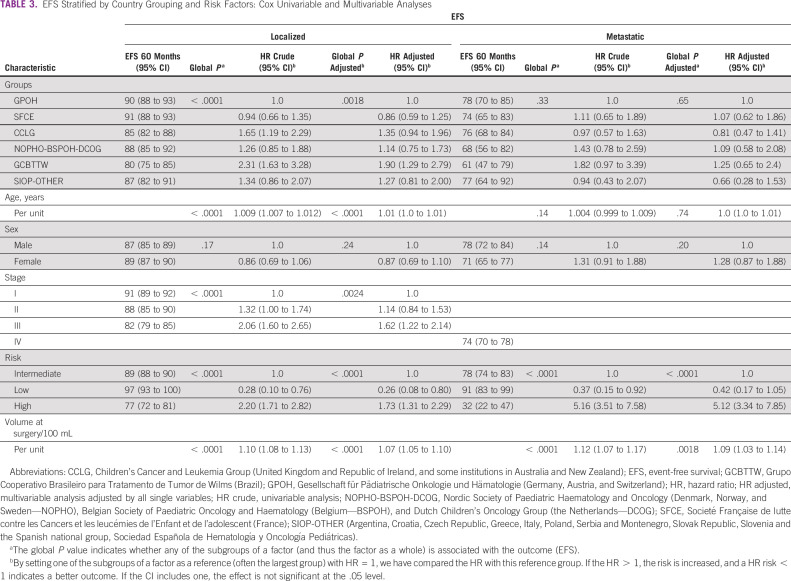
EFS Stratified by Country Grouping and Risk Factors: Cox Univariable and Multivariable Analyses

**TABLE 4:**
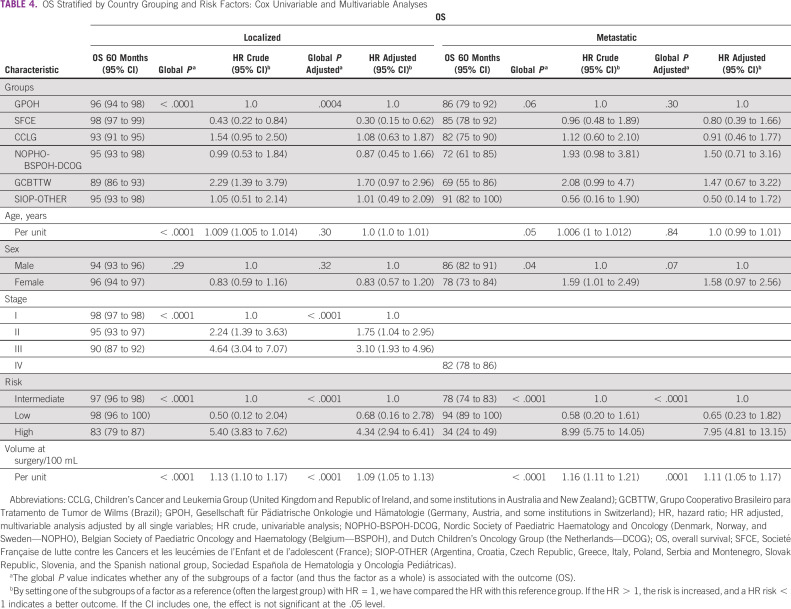
OS Stratified by Country Grouping and Risk Factors: Cox Univariable and Multivariable Analyses

Comparative analysis of OS showed the SFCE group had significantly better OS for localized disease than the other groups (*P* = .001). Stage II (*P* = .04) and III (*P* < .0001), high-risk tumors (*P* < .0001), and tumor volume at surgery (*P* < .0001) were associated with increased risk of death. Only high-risk tumors (*P* < .0001) and tumor volume at surgery (*P* < .0001) were significantly associated with an increased risk of death in MVA for metastatic disease (Table 4).

### Toxicity

Death by treatment-related toxicity differed between country groups. The GCBTTW group had the highest percentage of deaths from toxicity (12.2%). Three children died of second cancers (two acute myeloid leukemia and one osteosarcoma; Table 5).

**TABLE 5:**
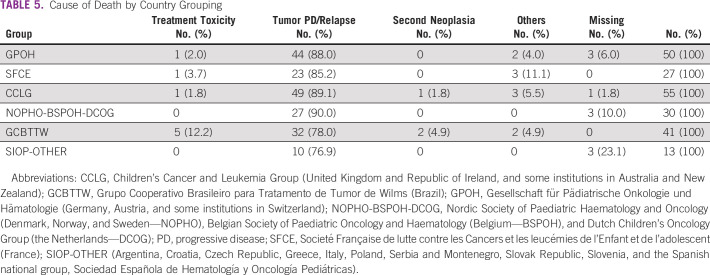
Cause of Death by Country Grouping

## DISCUSSION

Since 1971, seven studies and trials conducted by the SIOP-RTSG (formerly known as SIOP Nephroblastoma Group) have contributed to improvements in the diagnostics and management of WT.^15^ In the most recent completed trial (SIOPWT2001) reported here, the number of participating centers expanded to 251 from 26 countries, including Brazil, a large upper-middle–income country.^13^ The SIOPWT2001 study protocol has been adapted for use in several low- and middle-income countries to improve survival chances in these settings.^16,17^ To our knowledge, however, this is the first time that the SIOP-RTSG has performed an internal comparative analysis of its own study database to look for and understand the basis of any geographical variations to support future research and improvement work.

The analysis presented here found differences in clinical demographics, treatment, and survival outcomes among geographic populations who registered patients in SIOPWT2001 trial and study. Many potential reasons for these observed variations are considered.

There was a significant difference in the distribution of stages I, II, or III among children with localized WTs by country group (*P* < .001). This was especially notable comparing the proportions with stage I WT between GPOH and CCLG (53.4% *v* 33.5%, respectively). CCLG also has a higher mean tumor volume at diagnosis. Pritchard-Jones et al^18^ have suggested that these differences could be explained by distinct characteristics of national health care systems, with fewer cases diagnosed incidentally in the United Kingdom compared with Germany.

There was also variation in the proportion of patients with metastatic disease at diagnosis, ranging from 11.1% (Brazil) to 18.2% (CCLG), with the other European regions having intermediate proportions (GPOH 15.6%, SFCE 14.6%, and NOPHO-BSPOH-DCOG 14.1%). This variation may be partially explained by the sensitivity of the imaging modalities used for detection of metastases. The SIOPWT2001 trial and study required a chest X-ray in a posteroanterior and lateral view and although chest CT was common practice, it was not mandatory. The exact number of patients who had chest CT at diagnosis was not fully documented. The apparent lower proportion of patients presenting with metastases in Brazil could be explained by greater reliance on chest X-rays for staging purposes compared with other groups. Chest X-rays are known to reliably show nodules 1 cm or more in diameter, and chest CT has a higher sensitivity for detection of smaller lesions (< 1 cm).^19^ Analysis of SIOPWT2001 data has shown that survival of patients with CT-only detected lung lesions (typically 3-10 mm) was inferior to that of true localized-disease patients and superior to that of patients with metastatic disease.^20^ A further reason for the low proportion of patients with stage IV in Brazil is due to the fact that not all Brazilian centers registered their metastatic patients. Some institutions registered only cases that were randomized (stage II or III, intermediate-risk WT; B. de Camargo, personal communication, September 2021).

An important aspect of the SIOP-RTSG studies is the availability of central pathology review (CPR). This has revealed discrepancies between local pathology report and CPR regarding staging of localized disease in 9%-18% of cases.^21,22^ The most frequent discrepancy was between stage I and II. This fact needs to be considered when interpreting the stage distribution in our current analysis. This also has an impact on treatment intensity and may influence outcome.

CPR is important for standardization of histologic risk group classification. The frequency of discrepancies in histologic subtype has varied from 14% in SIOP 93-01^23^ to 23% in SIOPWT2001 (unpublished data). Perhaps, most important in this regard is the recognition of high-risk tumors in time to influence risk-stratified treatment. Anaplastic WT is known to be inconsistently recognized, with 39% of anaplastic cases not identified by the local pathologists in the NWTSG-5 study.^24^ The lower percentage of cases that underwent CPR by GCBTTW may explain the low percentage of diffuse anaplasia (6/332 = 1.8%) reported by this group, although it is also possible that tumor biology may vary between populations. Future studies will provide better insight.

Imaging at diagnosis and to assess response to preoperative chemotherapy together with precise pathology diagnostics are essential components of accurate risk stratification to determine the preoperative and postoperative treatment. Incorrect diagnosis might result in undertreatment or overtreatment. Undertreated patients are at risk of relapse, and overtreatment may cause acute toxicities and increased risk of late effects.

There were differences in the proportion of children with localized tumors whose postoperative chemotherapy included doxorubicin, ranging from 37.5% (GPOH) to 48.5% (SFCE). This variation was not entirely explained by the different stage distribution between the groups. Use of the more intensive 4-drug regimen in patients whose metastases did not achieve complete remission with chemotherapy and/or surgery also showed considerable variation, with only 25.7% in GPOH compared with 36.4%-43.7% in all other groups. This could be due to true differences in burden of metastatic disease at diagnosis and its chemosensitivity, or due to differences in metastatic response assessment and decision making at multidisciplinary team meetings about escalation of chemotherapy versus surgical intervention.^25^

The most important adverse independent risk factors (MVA) for both EFS and OS were high-risk histology, tumor stage, and tumor volume after preoperative chemotherapy. The survival differences among country groups may be explained by variations in clinical demographics, tumor characteristics, and treatment. Slightly better OS was seen in SFCE. This might be explained by their more frequent use of doxorubicin-containing regimens in postoperative therapy of localized disease, although this may also be entirely because of appropriate use of risk-stratified regimen selection (Table 2). An alternative explanation could be more aggressive retreatment of relapse. The lowest survival in GCBTTW may be explained by underdiagnosis of metastatic disease and high-risk tumors because of lack of CPR.

Treatment-related deaths were more common in GCBTW than in other groups (12.2% *v* 0%-3.7%, respectively). Health care infrastructure, supportive care, and patient status (malnutrition and coinfections) might contribute to high levels of toxic deaths in middle-income countries.^26^

Our study presented several limitations. It was a retrospective analysis, but on the basis of a large cohort of prospectively registered patients. Data on radiotherapy were not available for all patients, which precluded any conclusions on the relative contribution of that treatment modality. There was a lack of information on variation in protocol adherence and possible association with outcomes and the impact of genetic background and molecular characteristics of the tumors on clinical characteristics.

The population studied from the GCBTTW group may be somewhat biased. Brazil is an upper-middle-income country that enrolled only a minority (approximately 10%, 332 patients) of the estimated national population incidence over the 10-year study duration (estimated number of children younger than 14 years with renal tumors per year: 369).^27,28^ The Western European countries generally enrolled > 90% of population incident cases. Barriers to trial participation are numerous and challenging (mainly in low-income and middle-income countries). The positive impact of participation in clinical trials on improvement in cancer survival rates more generally is well known, but unfortunately, < 20% of children with cancer worldwide benefit from large cooperative group clinical research efforts.^29^

In conclusion, this study demonstrates that there are international differences in patients with WT with regards to histology, stage distribution, applied treatments, and survival rates. These comparative analyses highlight possible areas for health care and infrastructural improvements that may enable earlier diagnosis and enhanced standardization of risk stratification. The participation of the Brazilian GCBTTW group in the SIOPWT2001 trial and study has facilitated international benchmarking of factors influencing survival rates in a relatively resource-limited country. The ongoing SIOP–RTSG 2016 UMBRELLA study has the aim of international harmonization of definitions, diagnosis, treatment, and radiology and pathology review in combination with identification of novel adverse genetic signatures.^30^ Providing access to centralized expertise for radiology and pathology review is a priority in the current protocol.

## References

[1] Allemani C, Matsuda T, Di Carlo V, et al: Global surveillance of trends in cancer survival 2000-14 (CONCORD-3): Analysis of individual records for 37 513 025 patients diagnosed with one of 18 cancers from 322 population-based registries in 71 countries. Lancet 391:1023-1075, 2018 29395269 10.1016/S0140-6736(17)33326-3 PMC5879496

[2] Gatta G, Botta L, Capocaccia R, et al: Staging childhood cancers in Europe: Application of the Toronto stage principles for neuroblastoma and Wilms tumour. The JARC pilot study. Pediatr Blood Cancer 68:e29020, 2021 34114308 10.1002/pbc.29020

[3] Atun R, Bhakta N, Denburg A, et al: Sustainable care for children with cancer: A Lancet Oncology Commission. Lancet Oncol 21:e185-e224, 2020 32240612 10.1016/S1470-2045(20)30022-X

[4] Denburg A, Rodriguez-Galindo C, Joffe S: Clinical trials infrastructure as a quality improvement intervention in low- and middle-income countries. Am J Bioeth 16:3-11, 2016 10.1080/15265161.2016.1170230 27216089

[5] Nakata K, Colombet M, Stiller CA, et al: Incidence of childhood renal tumours: An international population-based study. Int J Cancer 147:3313-3327, 2020 32902866 10.1002/ijc.33147 PMC7689773

[6] Nakata K, Williams R, Kinoshita Y, et al: Comparative analysis of the clinical characteristics and outcomes of patients with Wilms tumor in the United Kingdom and Japan. Pediatr Blood Cancer 68:e29143, 2021 34056846 10.1002/pbc.29143

[7] Easton JC, Gomez S, Asdahl PH, et al: Survival of high-risk pediatric neuroblastoma patients in a developing country. Pediatr Transplant 20:825-830, 2016 27235336 10.1111/petr.12731 PMC5661966

[8] Castellanos EM, Barrantes JC, Báez LF, et al: A chemotherapy only therapeutic approach to pediatric Hodgkin lymphoma: AHOPCA LH 1999. Pediatr Blood Cancer 61:997-1002, 2014 24347509 10.1002/pbc.24905

[9] Bouda GC, Traoré F, Couitchere L, et al: Advanced Burkitt lymphoma in sub-Saharan Africa pediatric units: Results of the third prospective multicenter study of the Groupe Franco-Africain d'Oncologie Pédiatrique. JCO Glob Oncol 5:1-9, 2019 10.1200/JGO.19.00172 PMC6939747 31794283

[10] Brunetto AL, Castillo LA, Petrilli AS, et al: Carboplatin in the treatment of Ewing sarcoma: Results of the first Brazilian collaborative study group for Ewing sarcoma family tumors-EWING1. Pediatr Blood Cancer 62:1747-1753, 2015 25917418 10.1002/pbc.25562

[11] Luna-Fineman S, Chantada G, Alejos A, et al: Delayed enucleation with neoadjuvant chemotherapy in advanced intraocular unilateral retinoblastoma: AHOPCA II, a prospective, multi-institutional protocol in Central America. J Clin Oncol 37:2875-2882, 2019 31536438 10.1200/JCO.18.00141 PMC6823891

[12] Olson TA, Murray MJ, Rodriguez-Galindo C, et al: Pediatric and adolescent extracranial germ cell tumors: The road to collaboration. J Clin Oncol 33:3018-3028, 2015 26304902 10.1200/JCO.2014.60.5337 PMC4979195

[13] Pritchard-Jones K, Bergeron C, de Camargo B, et al: Omission of doxorubicin from the treatment of stage II-III, intermediate-risk Wilms' tumour (SIOP WT 2001): An open-label, non-inferiority, randomised controlled trial. Lancet 386:1156-1164, 2015 26164096 10.1016/S0140-6736(14)62395-3

[14] Vujanić GM, Sandstedt B, Harms D, et al: Revised International Society of Paediatric Oncology (SIOP) working classification of renal tumors of childhood. Med Pediatr Oncol 38:79-82, 2002 11813170 10.1002/mpo.1276

[15] Graf N, Bergeron C, Brok J, et al: Fifty years of clinical and research studies for childhood renal tumors within the International Society of Pediatric Oncology (SIOP). Ann Oncol 32:1327-1331, 2021 34416363 10.1016/j.annonc.2021.08.1749

[16] Chagaluka G, Paintsil V, Renner L, et al: Improvement of overall survival in the Collaborative Wilms Tumour Africa Project. Pediatr Blood Cancer 67:e28383, 2020 32391983 10.1002/pbc.28383

[17] Shyirambere C, Xu MJ, Elmore SN, et al: Treating nephroblastoma in Rwanda: Using International Society of Pediatric Oncology guidelines in a novel oncologic care model. J Glob Oncol 2:105-113, 2016 28717689 10.1200/JGO.2015.000067 PMC5495448

[18] Pritchard-Jones K, Graf N, van Tinteren H, et al: Evidence for a delay in diagnosis of Wilms' tumour in the UK compared with Germany: Implications for primary care for children. Arch Dis Child 101:417-420, 2016 26948824 10.1136/archdischild-2015-309212 PMC4862069

[19] Grundy PE, Green DM, Dirks AC, et al: Clinical significance of pulmonary nodules detected by CT and not CXR in patients treated for favorable histology Wilms tumor on national Wilms tumor studies-4 and -5: A report from the Children's Oncology Group. Pediatr Blood Cancer 59:631-635, 2012 22422736 10.1002/pbc.24123 PMC3397278

[20] Smets AMJB, van Tinteren H, Bergeron C, et al: The contribution of chest CT-scan at diagnosis in children with unilateral Wilms' tumour. Results of the SIOP 2001 study. Eur J Cancer 48:1060-1065, 2012 21703848 10.1016/j.ejca.2011.05.025

[21] de Kraker J, Delemarre JF, Lilien MR, et al: Misstaging in nephroblastoma. Causes and consequences. A report of the Sixth Nephroblastoma Trial and Study of the International Society of Paediatric Oncology. Eur J Pediatr Surg 9:153-157, 1999 10427490 10.1055/s-2008-1072232

[22] Vujanić GM, Sandstedt B, Kelsey A, et al: Central pathology review in multicenter trials and studies: Lessons from the nephroblastoma trials. Cancer 115:1977-1983, 2009 19241454 10.1002/cncr.24214

[23] de Kraker J, Graf N, van Tinteren H, et al: Reduction of postoperative chemotherapy in children with stage I intermediate-risk and anaplastic Wilms' tumour (SIOP 93-01 trial): A randomised controlled trial. Lancet 364:1229-1235, 2004 15464183 10.1016/S0140-6736(04)17139-0

[24] Dome JS, Cotton CA, Perlman EJ, et al: Treatment of anaplastic histology Wilms' tumor: Results from the fifth National Wilms' Tumor Study. J Clin Oncol 24:2352-2358, 2006 16710034 10.1200/JCO.2005.04.7852

[25] Warmann SW, Furtwängler R, Blumenstock G, et al: Tumor biology influences the prognosis of nephroblastoma patients with primary pulmonary metastases: Results from SIOP 93-01/GPOH and SIOP 2001/GPOH. Ann Surg 254:155-162, 2011 21670612 10.1097/SLA.0b013e318222015e

[26] Israels T, Moreira C, Scanlan T, et al: SIOP PODC: Clinical guidelines for the management of children with Wilms tumour in a low income setting. Pediatr Blood Cancer 60:5-11, 2013 23015404 10.1002/pbc.24321

[27] Steliarova-Foucher E, Colombet M, Ries LAG, et al: International incidence of childhood cancer, 2001-10: A population-based registry study. Lancet Oncol 18:719-731, 2017 28410997 10.1016/S1470-2045(17)30186-9 PMC5461370

[28] World Population Prospects—Population Division—United Nations. https://population.un.org/wpp/

[29] Rodriguez-Galindo C, Friedrich P, Alcasabas P, et al: Toward the cure of all children with cancer through collaborative efforts: Pediatric oncology as a global challenge. J Clin Oncol 33:3065-3073, 2015 26304881 10.1200/JCO.2014.60.6376 PMC4979198

[30] van den Heuvel-Eibrink MM, Hol JA, Pritchard-Jones K, et al: Position paper: Rationale for the treatment of Wilms tumour in the UMBRELLA SIOP-RTSG 2016 protocol. Nat Rev Urol 14:743-752, 2017 29089605 10.1038/nrurol.2017.163

